# Antibiofilm and staphyloxanthin inhibitory potential of terbinafine against *Staphylococcus aureus*: in vitro and in vivo studies

**DOI:** 10.1186/s12941-022-00513-7

**Published:** 2022-05-30

**Authors:** Momen Askoura, Nehal Yousef, Basem Mansour, Fatma Al-zahraa A. Yehia

**Affiliations:** 1 Department of Microbiology and Immunology, Faculty of Pharmacy, Zagazig University, Zagazig, 44519 Egypt; 2 Department of Pharmaceutical Chemistry, Faculty of Pharmacy, Delta University for Science and Technology, Gamasa 11152, Belqas, Egypt

**Keywords:** *Staphylococcus aureus*, Terbinafine, Staphyloxanthin, Biofilm, Virulence

## Abstract

**Background:**

Antimicrobial resistance is growing substantially, which necessitates the search for novel therapeutic options. Terbinafine, an allylamine antifungal agent that exhibits a broad spectrum of activity and is used in the treatment of dermatophytosis, could be a possible option to disarm *S. aureus* virulence.

**Methods:**

Terbinafine inhibitory effect on staphyloxanthin was characterized by quantitative measurement of staphyloxanthin intermediates and molecular docking. The effect of terbinafine on *S. aureus* stress survival was characterized by viable counting. The anti-biofilm activity of terbinafine on *S. aureus* was assessed by the crystal violet assay and microscopy. Changes in *S. aureus* membrane following treatment with terbinafine were determined using Fourier transform infrared (FTIR) analysis. The synergistic action of terbinafine in combination with conventional antibiotics was characterized using the checkerboard assay. qRT-PCR was used to evaluate the impact of terbinafine on *S. aureus* gene expression. The influence of terbinafine on *S. aureus* pathogenesis was investigated in mice infection model.

**Results:**

Terbinafine inhibits staphyloxanthin biosynthesis through targeting dehydrosqualene desaturase (CrtN). Docking analysis of terbinafine against the predicted active site of CrtN reveals a binding energy of − 9.579 kcal/mol exemplified by the formation of H-bonds, H-arene bonds, and hydrophobic/hydrophilic interactions with the conserved amino acids of the receptor pocket. Terbinafine treated *S. aureus* was more susceptible to both oxidative and acid stress as well as human blood killing as compared to untreated cells. Targeting staphyloxanthin by terbinafine rendered *S. aureus* more sensitive to membrane acting antibiotics. Terbinafine interfered with *S. aureus* biofilm formation through targeting cell autoaggregation, hydrophobicity, and exopolysaccharide production. Moreover, terbinafine demonstrated a synergistic interaction against *S. aureus* when combined with conventional antibiotics. Importantly, terbinafine attenuated *S. aureus* pathogenesis using mice infection model. qRT-PCR revealed that terbinafine repressed expression of the transcriptional regulators *sigB*, *sarA,* and *msaB*, as well as *icaA* in *S. aureus*.

**Conclusions:**

Present findings strongly suggest that terbinafine could be used safely and efficiently as an anti-virulent agent to combat *S. aureus* infections.

## Background

*Staphylococcus aureus* is a commensal bacterium that asymptomatically colonizes humans. However, *S. aureus* could be an extremely versatile pathogen in humans, causing life-threatening infections. *S. aureus* possesses a wide range of virulence factors that enable it to thrive within the host [1]. Diseases caused by *S. aureus* are diverse, ranging from minor skin infections to highly severe infections including pneumonia, endocarditis, and sepsis [2]. *S. aureus* infections are problematic because of the frequent emergence of antibiotic resistant strains of *S. aureus* [3]. The emergence of methicillin-resistant *S. aureus* (MRSA) and vancomycin-resistant MRSA (VRSA) limits the usage of antibiotics and renders treatment more challenging [4]. In view of these facts, new antibacterial agents are urgently needed that target *S. aureus* with no pressure on the bacterium in order to minimize the development of antibiotic resistance [5].

Staphyloxanthin is considered the hallmark virulence factor of *S. aureus* [6]. Staphyloxanthin is a golden-yellow carotenoid pigment found in the plasma membrane and acts as an antioxidant against host immune response. Furthermore, staphyloxanthin maintains the structural integrity of the bacterial membrane and is associated with bacterial survival under stressful conditions [7, 8]. Thus, disrupting staphyloxanthin biosynthesis has become an innovative anti-infective approach to alter *S. aureus* virulence with the advantage of minimizing the emergence of antibiotic resistant strains [9]. Several enzymes are involved in staphyloxanthin biosynthesis. First, CrtM, a dehydrosqualene synthase, catalyzes the condensation of two molecules of farnesyl diphosphate to form 4,4′-diapophytoene, which is further dehydrogenated to 4, 4′-diaponeurosporene by CrtN, a dehydrosqualene desaturase. Finally, 4, 4′-diaponeurosporene undergoes oxidation, glycosylation, and esterification to give staphyloxanthin [10].

In addition to staphyloxanthin and its role in staphylococcal pathogenesis, *S. aureus* forms biofilm, which is an adherent microbial community on both biotic and abiotic surfaces [11]. Biofilm formation is mediated by self-secreted polymeric substances that provides a hydrated matrix structure that protects bacterial cells against both antimicrobials and host immune response [12]. Biofilm-related infections account for increased morbidity and mortality and could result in prolonged durations of hospitalization [13]. Therefore, the abolishment of bacterial biofilm formation has been reported to be an efficient strategy to counteract bacterial infections [14].

Naphthalene-containing compounds are known to have a variety of biological activities, including antimicrobial properties [15, 16]. The Food and Drug Administration has approved numerous naphthalene derivatives as therapeutics, such as naftifine and terbinafine. Terbinafine is an allylamine fungicidal, administered either topically or orally, and used in the treatment of superficial fungal infections of the skin and nails. Additionally, terbinafine has clinically relevant antibacterial properties that may be useful in mixed bacterial and fungal infections as athlete’s foot [17, 18]. Naftifine has been shown previously to have potent staphyloxanthin inhibiting activity [19]. Therefore, based on the structural similarity, the present study aims to characterize the anti-virulent potential of terbinafine against *S. aureus*. The influence of terbinafine on staphyloxanthin biosynthesis, biofilm formation, as well as *S. aureus* host pathogenesis will be fully uncovered herein.

## Materials and methods

### Bacterial strains and growth conditions

*Staphylococcus aureus* ATCC 6538 and five clinical isolates recovered from patients admitted to the Zagazig University Hospital in Egypt were included in this study. Clinical isolates were selected based on their pigment content; four isolates were pigmented, and one isolate was non-pigmented. Clinical *S. aureus* isolates were further characterized using 16S rRNA gene sequencing and results were deposited in GenBank (https://www.ncbi.nlm.nih.gov/) under accession numbers **ON032523, ON032524, ON032525, ON032526 and ON032527**. Bacteria were grown aerobically in a trypticase soy broth (TSB). For biofilm and virulence assays, bacterial cells were grown in TSB supplemented with 1% glucose (TSBG).

### Determination of the minimum inhibitory concentration (MIC) of terbinafine

The MIC of terbinafine (Mash Premiere, Egypt) against *S. aureus* ATCC 6538 was determined as described before [20]. Results were recorded and MIC values were determined in triplicate. The effect of terbinafine on *S. aureus* viability was determined by growing bacteria in the presence of sub-MIC of terbinafine (132, 164, and 328 µg/mL) and bacterial growth was compared with untreated bacteria. Similarly, the impact of terbinafine on *S. aureus* metabolic activity was assessed by the alamar blue assay as previously described [21]. Resazurin solution (6.5 mg/mL) was added to both terbinafine treated and untreated bacteria and incubated in the dark for 4 h. The fluorescence signals of the supernatant were recorded at 590 nm emission and 560 nm excitation wavelengths [21].

### Investigation of the inhibitory effect of terbinafine on staphyloxanthin biosynthesis.

Carotenoid pigment was extracted from both terbinafine treated and untreated *S. aureus* and estimated as described before [22]. *S. aureus* strains were grown in TSB with varying concentrations of terbinafine (3.28, 8.2, 16.4, 24.6, 32.8, and 49.2 µg/mL) at 37 °C for 24 h. Cells suspensions were adjusted to an optical density of 2 at 600 nm (4 × 10^9^ colony forming units (CFUs) in a 10 mL volume). Bacterial cells were harvested by centrifugation and resuspended in 99% methanol and agitated for 30 min at 55 °C in the dark. Following centrifugation, the supernatant absorbance was measured at 465 nm using a Bio-Tek synergy HT microplate reader, USA. The half maximal inhibitory concentration (IC_50_) of terbinafine was estimated as previously described [23]. Additionally, staphyloxanthin biosynthesis intermediates were determined in response to terbinafine treatment. The absorbance of methanol-extracted carotenoids containing staphyloxanthin intermediates was measured using a plate reader (Synergy HT, BioTek) at wavelengths 286 nm, 435 nm, 455 nm, and 465 nm for 4,4′-diapophytoene 4,4′-diaponeurosporene 4,4′diaponeurosporenic acid, and staphyloxanthin, respectively [24].

### Molecular docking analysis

The sequence of amino acids of CrtN (WP_000686169.1) was obtained as a primary structure in FASTA form from the NCBI database, and the predicted 3D structure of the receptor was obtained from the IntFOLD Server (Version 5.0) [25]. Terbinafine was drawn into the Marvin Sketch of Marvin site (http://www.chemaxon.com) to generate the lowest energy conformer. The Dock module of Molecular Operating Environment (MOE 2019.0102) was used in molecular docking analysis [26]. All hydrogen atoms with their standard geometry were inserted to the protein predicted 3D structure, and then their energy was minimized. Using the flexible ligand mode, terbinafine was docked into the rigid binding pocket of the protein. The placement phase generates poses from ligand conformations. The ligand free energy of binding from a predicted pose is calculated using the GBVI/WSA ΔG as a force field-based scoring function [27].

### Characterization of terbinafine impact on *S. aureus* susceptibility to H_2_O_2_, acid stress, and whole blood killing

The sensitivity of *S. aureus* to H_2_O_2_ oxidative stress, acid stress, and whole blood killing following treatment with terbinafine was determined and compared with untreated cells [28]. Briefly, *S. aureus* ATCC 6538 and the non-pigmented *S. aureus* isolate were cultured in TSB with sub-MIC of terbinafine (328 μg/mL). Bacterial pellets were collected and adjusted to a concentration of 10^7^ CFU/mL. For oxidative stress, H_2_O_2_ (1.5%) was added, and bacterial survival was assessed every 15 min over a 1-h period. For acid stress, *S. aureus* cells (10^6^ CFU/mL) were exposed to acid stress at pH 4 (adjusted with acetic acid) and bacterial survival was assessed. Bacterial survival to whole blood killing was evaluated as follows: freshly drawn heparinized human blood and *S. aureus* cultures (6 × 10^8^ CFU/mL) were mixed in 3:1 volume ratio and incubated for 2 h. For all experiments, bacterial viability was assessed by counting surviving bacteria after serial dilution in phosphate buffered saline (PBS) and plating on TSA and compared to control untreated cells.

### Fourier transform infrared (FTIR) spectroscopy analysis

Changes in *S. aureus* membrane upon terbinafine exposure were determined using FTIR analysis [29]. *S. aureus* ATCC 6538 overnight culture in the presence of terbinafine (328 µg/mL) was adjusted to 9 × 10^8^ CFU/mL. Bacterial suspension was centrifuged and collected cells were subjected to FTIR analysis (Bruker Alpha FTIR) and the spectrum was scanned in the range of 4000–500 cm^−1^. The FTIR spectra of treated cells were plotted as transmittance against wave number and compared to untreated *S. aureus*.

### Evaluation of the effect of terbinafine on* S. aureus *survival to polymyxin B and biofilm formation

The effect of terbinafine on *S. aureus* survival to the membrane acting antibiotic polymyxin B was determined. Overnight *S. aureus* cultures (5 × 10^6^ CFU/mL) in the presence of terbinafine were treated with 1 mM polymyxin B and incubated for 30 min. Bacterial viability was assessed by serial dilution and plating on TSA and compared with untreated bacteria [30]. The biofilm quantitative assay was performed as described before [31] using the crystal violet (CV) method. *S. aureus* ATCC 6538 (10^6^ CFU/mL) was allowed to form biofilm for 48 h. Formed biofilm was assayed by staining with CV solution and glacial acetic acid (33%) for CV solubilization. The absorbance was measured at 570 nm and the percentage of biofilm inhibition was calculated as follows: % of inhibition = [(Control OD_570 nm_−Treated OD_570 nm_)/Control OD_570 nm_] × 100. In addition, the inhibitory effect of terbinafine on *S. aureus* biofilm was assessed using both light and scanning electron microscopes [32]. Biofilms were developed on polystyrene discs in the presence and absence of terbinafine (132, 164 and 328 μg/mL) as described above. Discs were washed twice with PBS, fixed by 2.5% glutaraldehyde for 2 h, and dehydrated using ethanol. Finally, the discs were air dried and gold coated before imaging using JEOL scanning microscope (JSM-T100, Japan).

### Characterization of the influence of terbinafine on *S. aureus* auto-aggregation, surface hydrophobicity, exopolysacchraide (EPS) formation and cell autolysis

*Staphylococcus aureus* auto-aggregation assay was performed as reported earlier [33]. Overnight *S. aureus* ATCC 6538 culture in TSBG containing terbinafine was centrifuged and then bacteria were resuspended in PBS and allowed to stand at 37 °C for 20 h. Cell density of the upper portion of PBS containing cells was measured at OD_600_ in comparison with untreated cells. As described earlier [34], the bacterial surface hydrophobicity index was determined. *S. aureus* ATCC 6538 grown in TSBG containing terbinafine (132, 164, and 328 μg/mL) was collected by centrifugation. Bacterial suspensions were adjusted to initial absorbance (Ai = 1.0), toluene was added, vortexed, and the absorbance of aqueous phase (Af) was measured. The hydrophobicity index (HI) was expressed as: HI = (Ai−Af)/Ai × 100% and compared to the HI of control untreated bacteria. EPS production by *S*. *aureus* was quantified using the phenol–sulfuric acid method as previously mentioned [35]. Glass slides were immersed in TSBG containing *S. aureus* with and without terbinafine and incubated for 24 h. Glass slides were removed, washed, and formed cell suspensions were mixed with 5% phenol/H_2_SO_4_. The mixture was left for 1 h and the supernatant absorbance was measured at 490 nm. Finally, the impact of terbinafine on bacterial induced cell autolysis was characterized. *S. aureus* ATCC 6538 culture in TSB containing 1 M NaCl and terbinafine (328 µg/mL), adjusted at A_580_ of 0.7, was centrifuged and washed. Cell pellets were resuspended in autolysis buffer (50 mM Tris–HCl and 0.1% Triton X-100) and incubated. Bacterial autolysis was measured at A_580_ over 3 h, where the decrease in optical density indicates a higher cell death rate [36].

### Characterization of terbinafine effect on *S. aureus* deoxyribonuclease (DNase), esterase, and lipase activity

DNase agar and tween substrate plates were prepared, and after solidification, cups were cut into the agar medium. Supernatant of overnight cultures of terbinafine treated and untreated *S. aureus* were added into wells and incubated. DNase activity was observed as a zone of clearance around the well after addition of 1 N HCL, while a white precipitation zone appeared around wells boundary, indicative of esterase and lipase activity [37, 38].

### qRT-PCR analysis

The qRT-PCR was performed to investigate the impact of terbinafine on the expression of *S. aureus* genes; *sarA, icaA*, *icaR*, agrA, *crtM*, *crtN*, *sodA*, *sodM, katA*, *sigB*, *msaB*, and y*jbH*. Briefly, an overnight culture of *S. aureus* ATCC 6538 grown in the presence of terbinafine was subjected to RNA extraction using the TRIzol reagent. Extracted RNA was purified using the Qiagen RNeasy minikit and reverse-transcribed into single-stranded complementary DNA (cDNA) using the QuantiTect-Reverse Transcription Kit, and cDNA was amplified using Maxima SYBR Green/Fluorescein qPCR Master Mix. The expression level of tested genes was normalized to 16S rRNA, and the 2 ^−∆∆*CT*^ method was used to calculate relative gene expression [39]. Primers used in the current study are listed in Table 1 [40–46].

**Table 1:** Primers used for qPCR analysis [40–46]

Gene name	Primer sequence (5′-3′)
*crtM* (F) *crtM* (F)	GGTGTTGCTGGTACAGTAGGTGAAG GCAACGATTCACCAAGTCTTCTTGCG
*crtN* (F) *crtN* (R)	CAGTGATTGGTGCAGGTGTC CATACGCCCGCCTACATTAT
*katA* (F) *katA* (R)	AAAGGTTCTGGTGCATTTGG AACGCAAATCCTCGAATGTC
*sodA* (F) *sodA* (R)	TGC ACGCTTTGGTTCAGGTTGGG GCGCCAATGTAGTCAGGGCGTTTG
*sodM* (F) *sodM* (R)	CCGGAAGCGATGAGGATGTCAGTC TGCCCCACTGCGCTTTGATGT
*icaA* (F) *icaA* (R)	CTGGCGCAGTCAATACTATTTCGGGTGTCT GACCTCCCAATGTTTCTGGAACCAACATCC
*icaR* (F) *icaR* (R)	TGCTTTCAAATACCAACTTTCAAGA ACGTTCAATTATCTAATACGCCTG
*sarA* (F) *sarA* (R)	CAAACAACCACAAGTTGTTAAAGC TGTTTGCTTCAGTGATTCGTTT
*agrA* (F) *agrA* (R)	TGATAATCCTTATGAGGTGCTT CACTGTGACTCGTAACGAAAA
*sigB* (F) *sigB* (R)	CGTCTCGGAACATGTACACTCCAAG GTCCTTTGAACGGAAGTTTGAAGCC
*cspA* (*msaB*) (F) *cspA* (*msaB*) (R)	TTTATCGAAGTTGAAGGAGAAAATG ACTCAACAGCTTGACCTTCTTCTAA
*yjbH* (F) *yjbH* (R)	AAGCCCCTTCTCTCGTTTTC TTTAAAAGTTTTTCTGGCCATTC
16 s *rRNA* (F) 16 s *rRNA* (R)	ACTCCTACGGGAGGCAGCAG ATTACCGCGGCTGCTGG

*F* Forward, *R* Reverse

### Checkerboard assay

The checkerboard assay was performed to measure the synergy between terbinafine and antibiotics targeting *S. aureus*; ampicillin, cefotaxime, azithromycin, ciprofloxacin, and gentamycin [47]. The effect of the combination between terbinafine and selected antibiotics was evaluated by calculating the fractional inhibitory concentration index (FICI) [48] according to the following formula:

FIC of terbinafine = MIC terbinafine in combination/MIC of terbinafine alone; FIC of antibiotic = MIC of antibiotic in combination/MIC of antibiotic alone; hence FIC index (FICI) = FIC of terbinafine + FIC of antibiotic. “Synergy” was defined when FICI was ≤ 0.5; while “additive” in which 0.5 ≤ FICI ≤ 1.0; moreover “indifferent” when the FICI is between 1 and 4.

### In vivo characterization of the influence of terbinafine on* S. aureus *virulence using mice infection model

The impact of terbinafine on *S. aureus* pathogenesis was determined [49, 50]. Briefly, overnight cultures of *S. aureus* ATCC 6538 in the presence of terbinafine (treated cells) and non-pigmented *S. aureus* were adjusted to 2.5 × 10^7^ CFU/mL in PBS. Five mice groups, each containing six mice, were included in the experiment. Untreated, terbinafine-treated, and non-pigmented *S. aureus* were injected into the abdominal cavity of 3-week-old albino mice (the first, second, and third groups, respectively). As negative controls, uninjected mice (the fourth group) and PBS-injected mice (the fifth group) were included. Mice were sacrificed at 24 h post-infection and the spleen, liver, and kidney were recovered aseptically and employed for bacterial load determination. Results were represented and expressed as the mean (CFU/g) ± standard errors. In addition, fragments of organs were fixed in buffered formalin (10%) for histopathological examination. The statistical analysis was examined by Mann–Whitney U analysis (*P* < 0.05 is considered significant).

### Statistical analyses

All experiments were carried in triplicate and the findings were expressed as the mean ± standard error. Unless otherwise stated, statistical analyses were conducted with GraphPad Prism 5 software using Student t-tests or one-way ANOVA.

## Results

### Terbinafine inhibits the biosynthesis of staphyloxanthin at sub-MIC concentrations

**Fig. 1:**
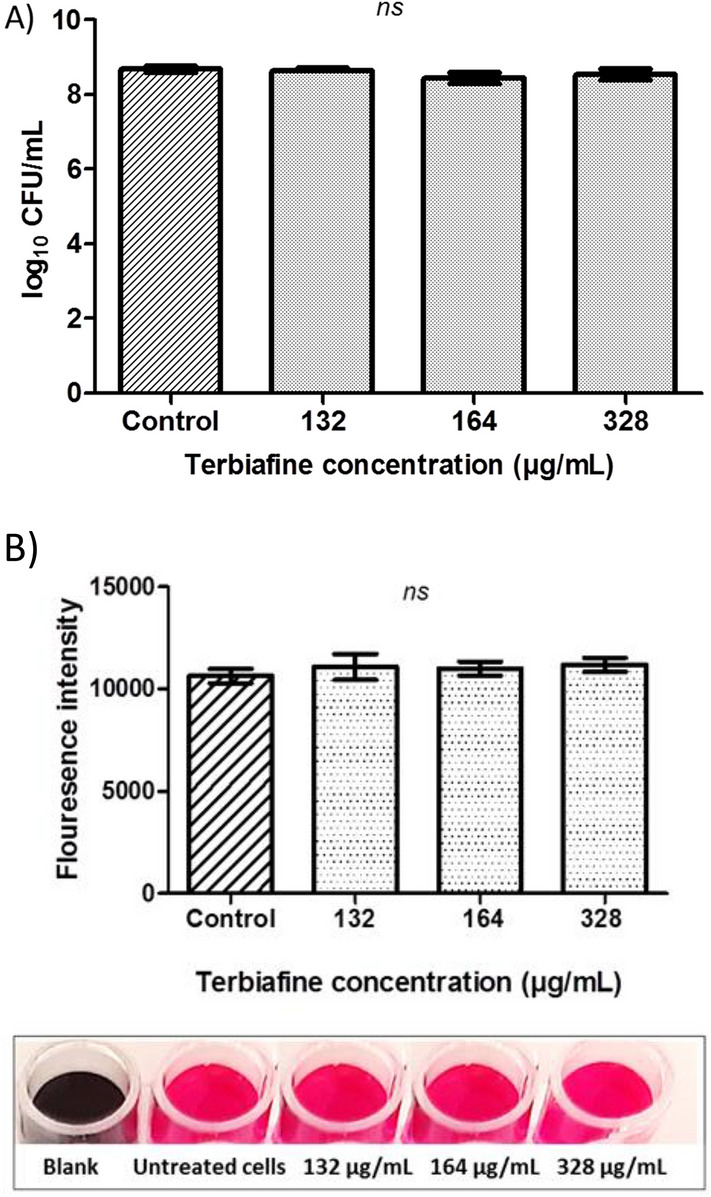
Terbinafine had no effect on *S. aureus* growth or viability at sub-MICs (132, 164, and 328 µg/mL). **A** CFU/mL of terbinafine treated cells and untreated cells with no significant difference, indicating that sub-MIC had non bactericidal activity. **B** Terbinafine treated cells were metabolically active as control cells, confirmed by the alamar blue assay. Data shown represent the mean ± standard error from triplicate experiments

**Fig. 2:**
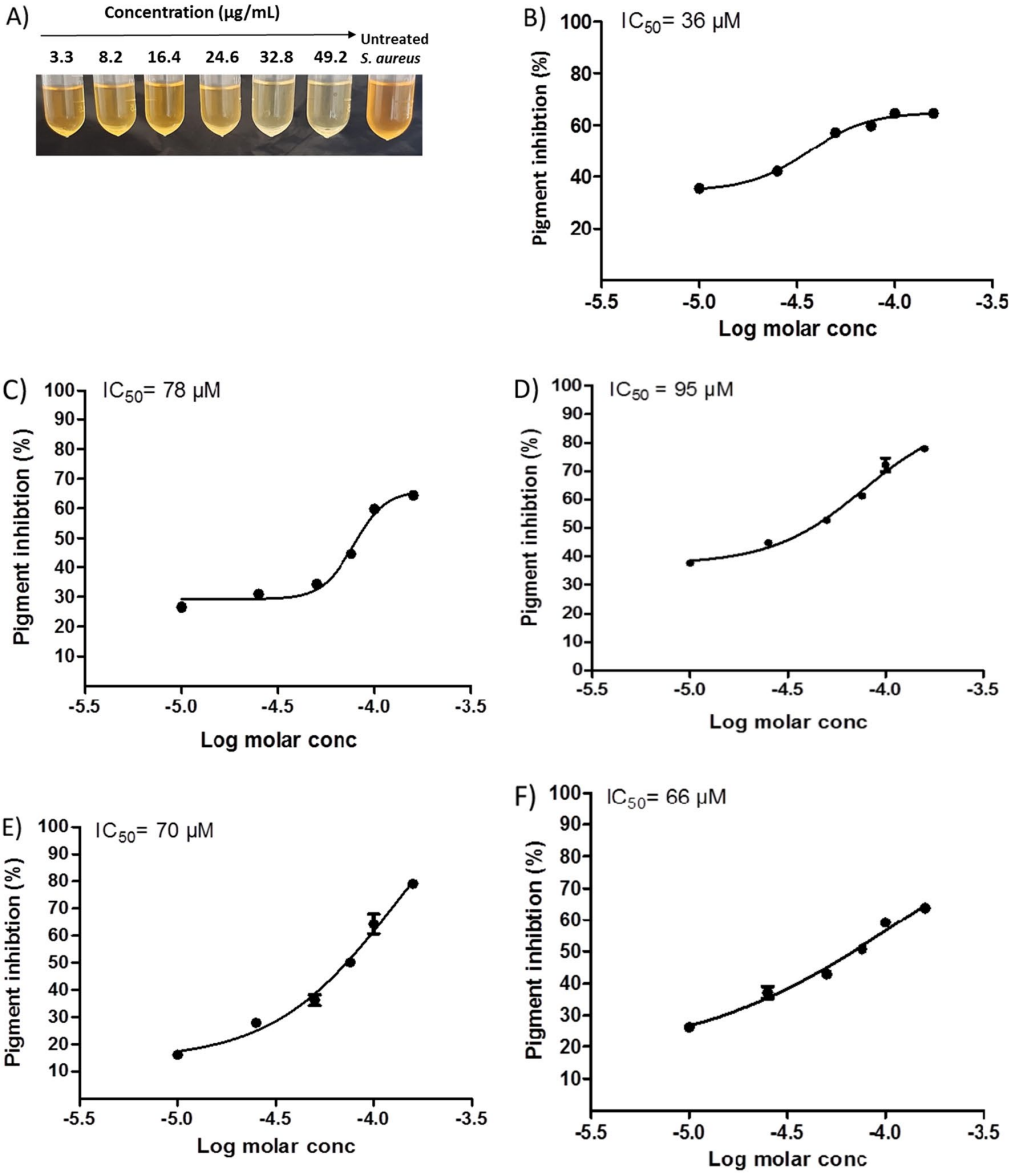
Terbinafine inhibited staphyloxanthin biosynthesis. **A** Terbinafine inhibition of *S. aureus* pigment in a dose dependent manner. **B** Dose inhibition curve with maximal inhibitory concentration (IC_50_) of terbinafine against *S. aureus* ATCC 6538. Dose inhibition curve with maximal inhibitory concentration (IC_50_) of terbinafine against *S. aureus* clinical isolates from wound infection (**C**), burn infection (**D**), respiratory tract infection (**E**) and urinary tract infection (**F**). Data shown represent the mean ± standard error from triplicate experiments

The MIC of terbinafine against *S. aureus* ATCC 6538 was determined as 2624 μg/mL. Results indicate that sub-MICs (132, 164, and 328 μg/mL) of terbinafine don’t interfere with *S. aureus* growth (Fig. 1A). Furthermore, the alamar blue assay shows that terbinafine-treated *S. aureus* was as metabolically active as untreated control cells (Fig. 1B). The inhibitory effect of terbinafine on staphyloxanthin biosynthesis in *S. aureus* ATCC 6538 was evaluated. Terbinafine inhibited staphyloxanthin biosynthesis in a dose dependent way with an IC_50_ of 36 µM (Fig. 2A–B). Similarly, terbinafine inhibited staphyloxanthin biosynthesis in *S. aureus* clinical isolates at micromolar concentrations (66–95 µM) (Fig. 2C–F).

### Terbinafine exhibits its activity via interference with CrtN

**Fig. 3:**
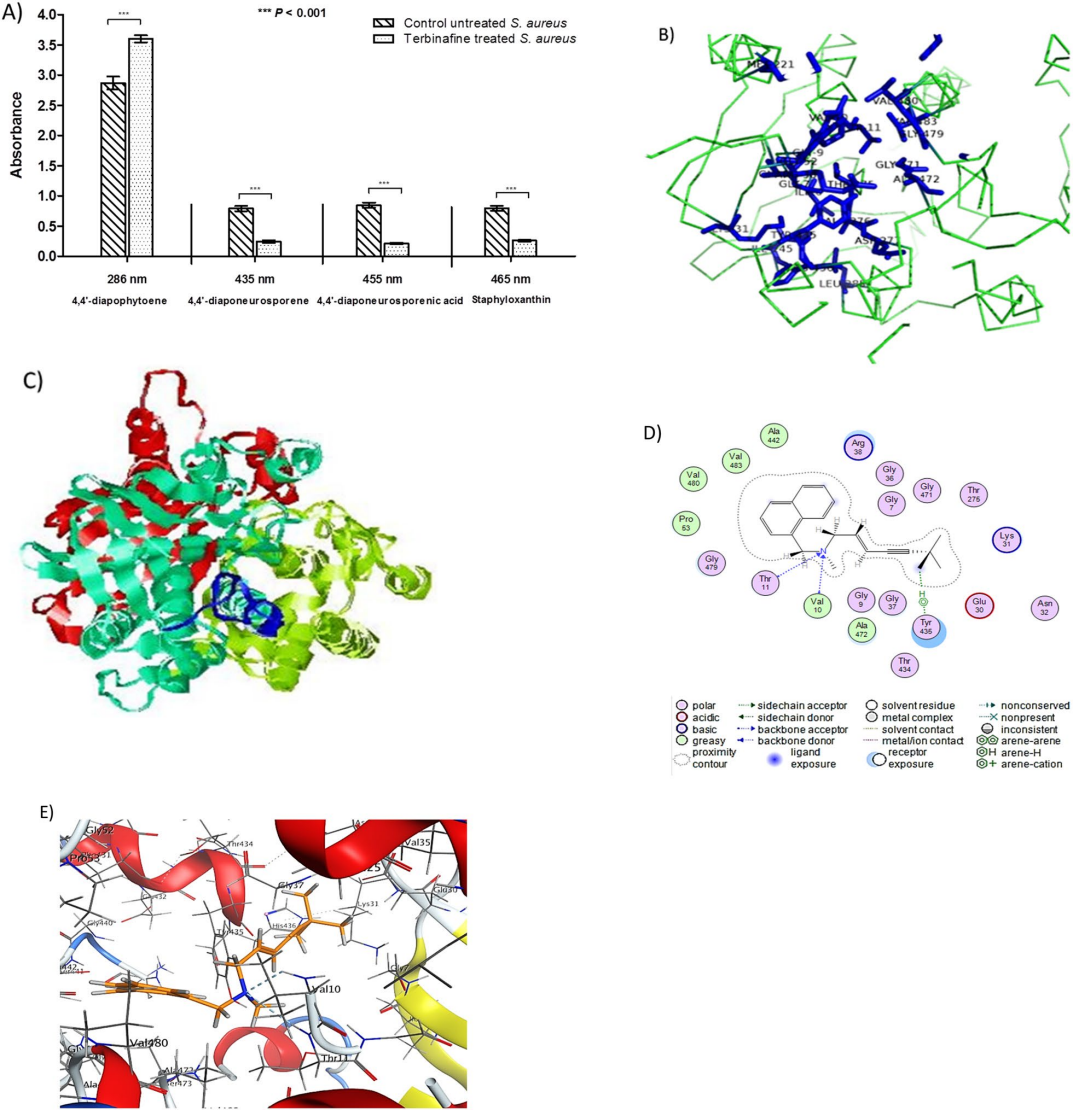
**A** Terbinafine treatment led to the accumulation of 4, 4′-diapophytoene. **B** Ligand binding residues prediction by for CrtN; predicted ligand binding residues are shown as blue sticks. **C** Domain boundary prediction for CrtN; the model was colored according to the predicted domains. The putative binding mode of terbinafine into the predicted active site of CrtN receptor in 2D (**D**) and 3D structure (**E**). The blue and cyan shadows of the ligand and active site amino acids, respectively, indicated strong hydrophobic/hydrophilic interactions. Data shown represent the mean ± standard of error from triplicate experiments. Using Student’s *t* test,* P* value < 0.05 was considered statistically significant

*Staphylococcus aureus* pigment methanolic extract was quantified spectrometrically upon treatment with sub-MIC of terbinafine. Importantly, treatment of *S. aureus* with terbinafine results in a significant accumulation of the CrtN substrate, 4,4′-diapophytoene. On the contrary, the amounts of subsequent staphyloxanthin biosynthesis intermediates (4,4′-diaponeurosporene and 4,4′-diaponeurosporenic acid) were significantly reduced in terbinafine treated *S. aureus* as compared to untreated bacteria (Fig. 3A). Furthermore, the molecular docking analysis validated terbinafine interaction with CrtN with a characteristic binding mode and affinity, exhibiting a binding energy of − 9.579 kcal/mol. Docking analysis results demonstrate that the tertiary amine group in the middle of terbinafine structure featured a conspicuous bifurcated H-bond with the conserved amino acids Val10 and Thr11 in the core of CrtN active site. Moreover, the flapping terminal of the ligand was fixed by an H-arene bond between *ter*.butyl group of the ligand and the conserved aromatic amino acid Tyr436 which enhanced the ligand/receptor complex stability. The overall recognition of the ligand inside the CrtN receptor hot spot was also augmented by the hydrophobic/hydrophilic interactions of the ligand and receptor (Fig. 3B–D).

### Terbinafine treated *S. aureus* is more susceptible to environmental stresses

**Fig. 4:**
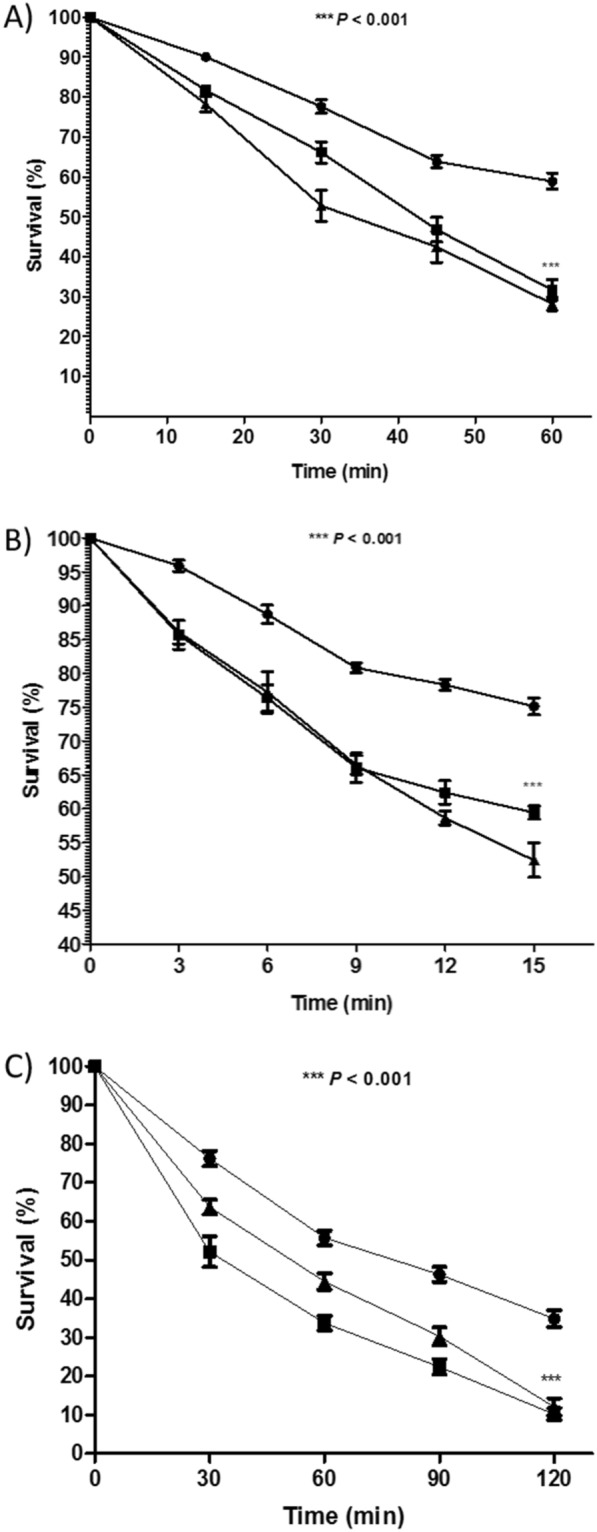
Terbinafine treatment senstised *S. aureus* ATCC 6538 to; **A** hydrogen peroxide, **B** acetic acid (pH = 4 ± 0.5) and **C** human blood cells. (•) untreated cells, (▲) terbinafine treated and (■) non-pigmented isolate. Data shown represent the mean ± standard of error from triplicate experiments. Using ANOVA test*, P* value < 0.05 was considered statistically significant

When compared to untreated *S. aureus*, terbinafine-treated *S. aureus* were more easily killed by hydrogen peroxide (bacterial survival of 28.1 ± 1.68% vs. 58.9% ± 2.01, respectively; Fig. 4A). Similarly, terbinafine treated *S. aureus* was more susceptible to acidic stress relative to untreated cells (bacterial survival of 52% ± 2.55 vs 75% ± 1.24, respectively; Fig. 4B). Importantly, terbinafine markedly sensitized *S. aureus* to human blood killing. The survival of the terbinafine treated *S. aureus* was almost three times lower than that of the untreated *S. aureus* (12.1% ± 2.194 vs. 34.8% ± 2.17, respectively; Fig. 4C).

### Terbinafine alters *S. aureus* membrane rigidity and increases bacterial susceptibility to membrane targeting antibiotics

**Fig. 5:**
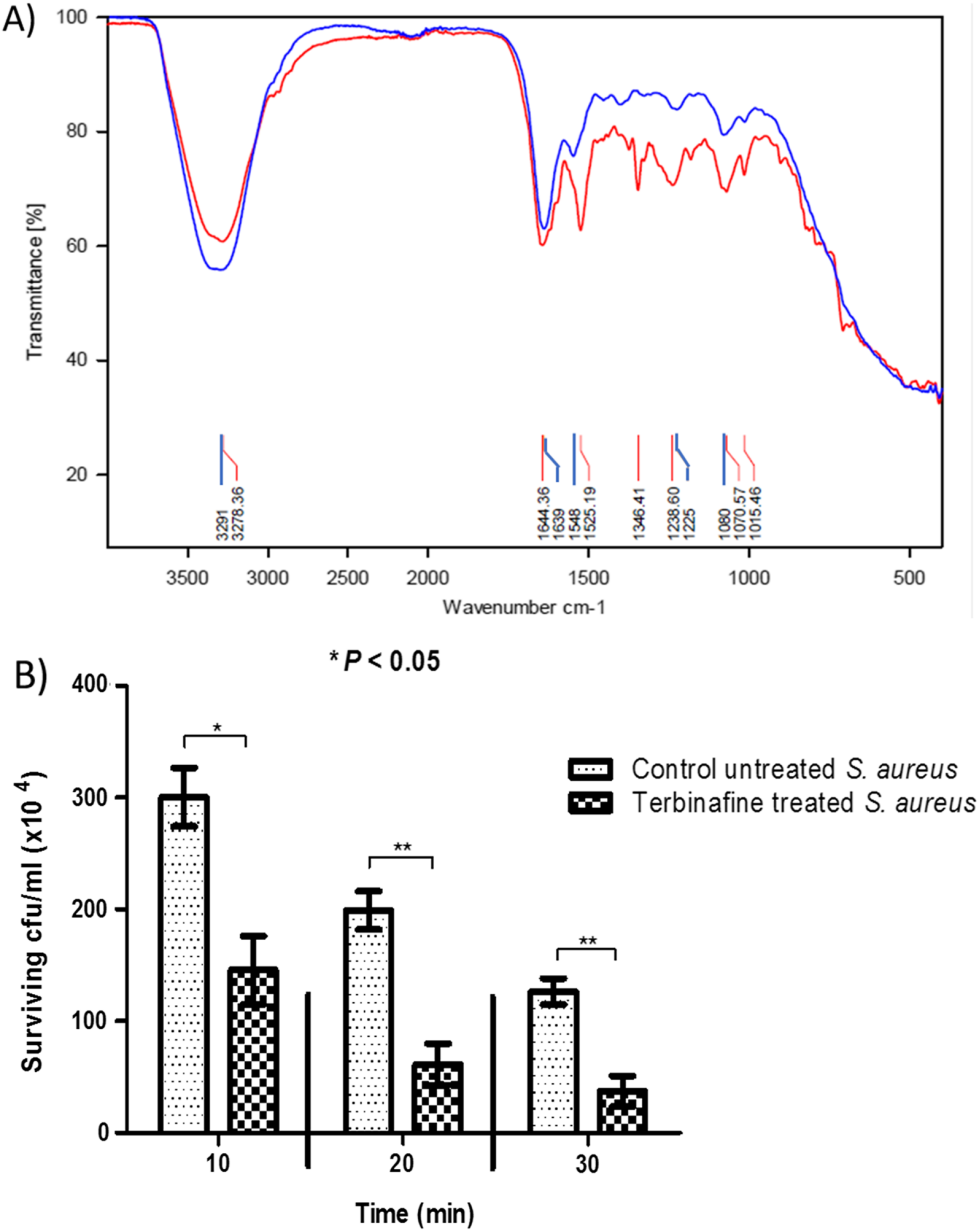
**A**) The FTIR spectra of *S. aureus* ATCC 6538 cells and terbinafine treated cells within wavenumber 4000–500 cm^−1^. Control untreated and terbinafine-treated cells are represented by red and blue lines, respectively. **B** Polymxin B survival assay indicating increased sensitivity of terbinafine treated cells to polymxin B than untreated cells. Data shown represent the mean ± standard of error from triplicate experiments. Using Student’s *t* test, *P* value < 0.05 was considered statistically significant

FTIR spectroscopic examination of terbinafine treated *S. aureus* revealed significant alterations in the spectrum profile as compared with untreated bacteria (Fig. 5A). The variation in bands corresponding to membrane phospholipids (1600–1200 cm^−1^) and polysaccharides (1100–1000 cm^−1^) confirmed the inhibitory effect of terbinafine on *S. aureus* staphyloxanthin. In support of these findings, terbinafine treated *S. aureus* showed a higher susceptibility to polymxin B than untreated cells, indicating alteration of bacterial membrane integrity upon bacterial treatment with terbinafine (Fig. 5B).

### Terbinafine possesses a potent anti-biofilm activity against *S. aureus*

**Fig. 6:**
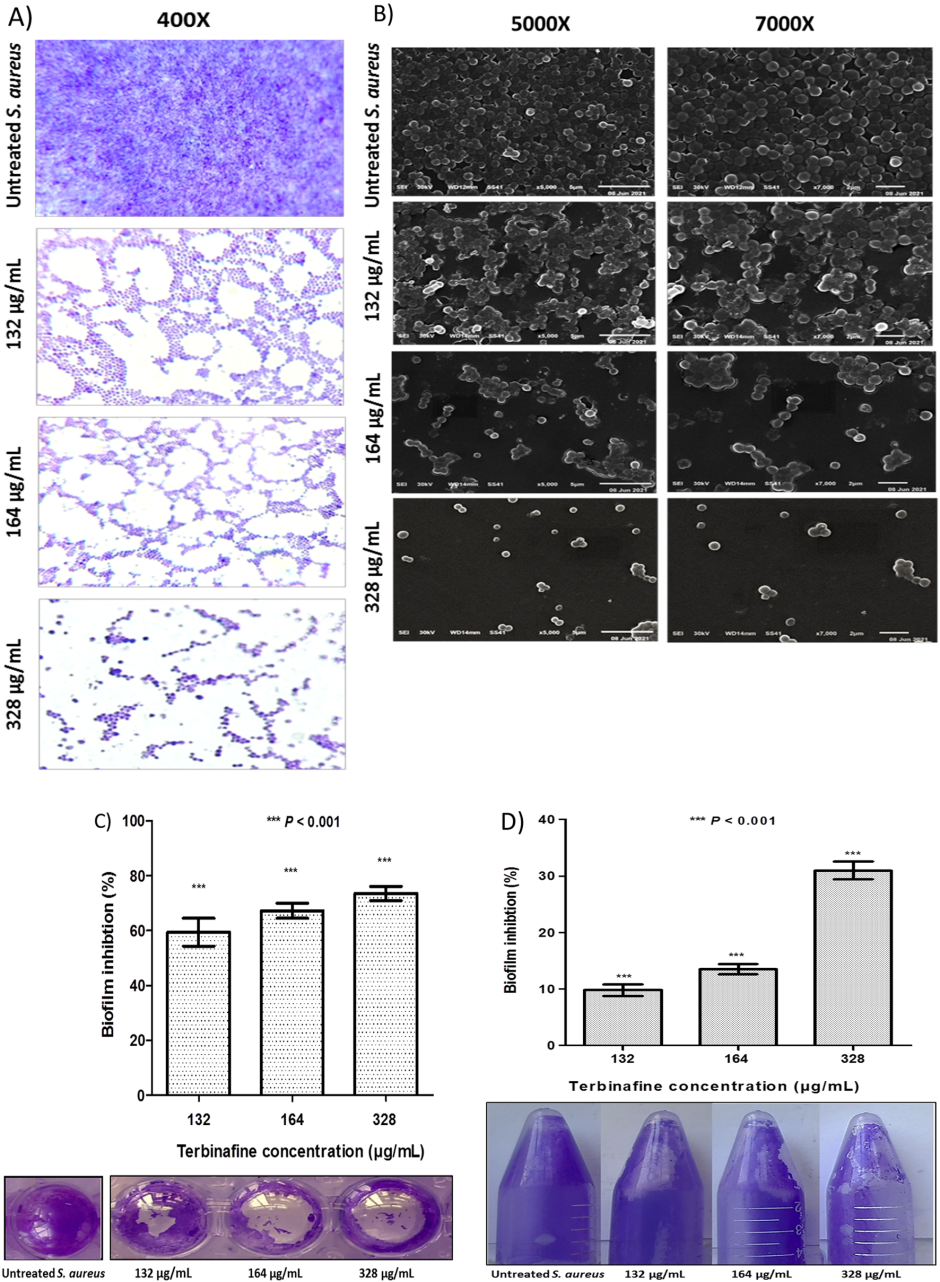
Terbinafine inhibited *S. aureus* biofilm formation in a dose dependent manner as observed from; light microscopic (**A**) and SEM (**B**) images. Quantitative crystal violet biofilm inhibition assay on; **C** polystyrene plate and **D** polypropylene tube upon terbinafine treatment. Data shown represent the mean ± standard of error from triplicate experiments. using Student’s *t* test, *P* value < 0.05 was considered statistically significant

Light and scanning electron microscopy (SEM) assessed the antibiofilm potential of terbinafine against *S. aureus*. Both light microscopy and SEM images of untreated cells revealed a firm established biofilm with a multilayer of aggregates and adherent cells (Fig. 6A, B, respectively). However, terbinafine treated cells showed a loose biofilm with scattered cells. Additionally, terbinafine showed a dose dependent biofilm inhibition on both polystyrene and polypropylene surfaces (Fig. 6C, D, respectively).

### Terbinafine diminishes cell auto-aggregation, surface hydrophobicity, and EPS production in* S. aureus *and enhances bacterial autolysis

**Fig. 7:**
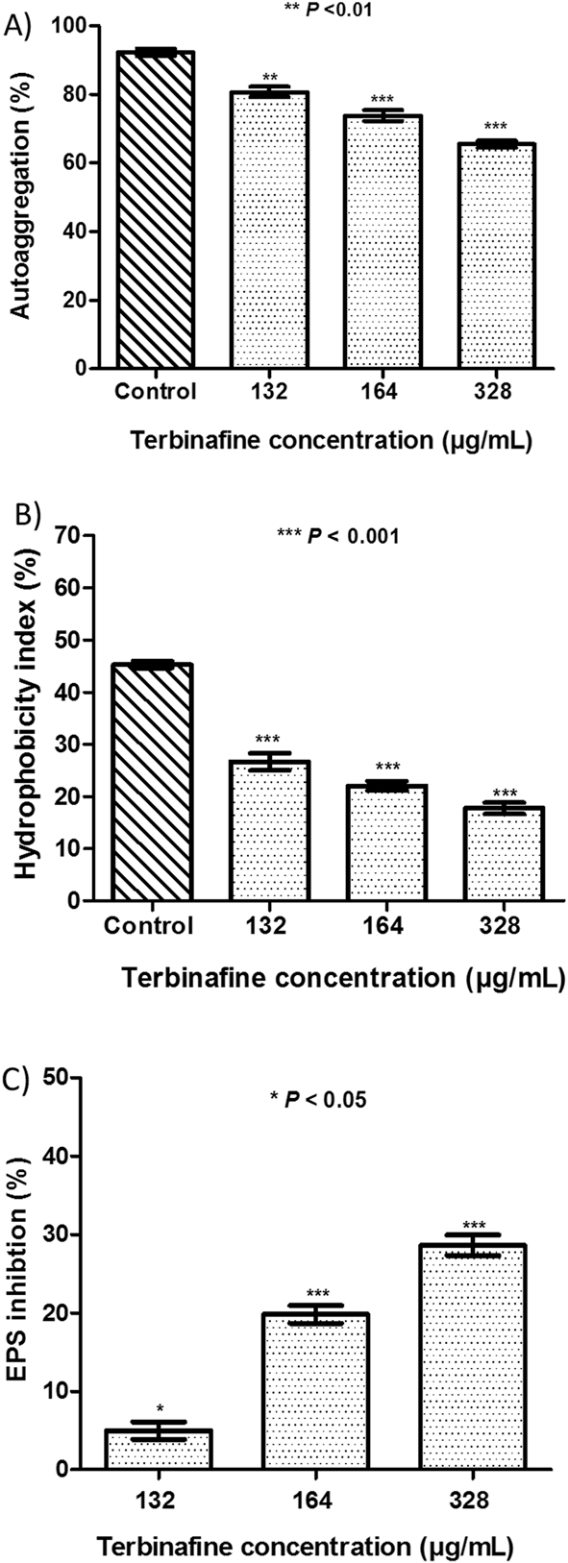
**A** Reduced auto-aggregation in terbinafine treated *S. aureus* ATCC 6538 in a dose dependent manner. **B** Hydrophobicity index of terbinafine treated cells was reduced in a dose dependent manner. **C** Inhibition of EPS in terbinafine treated cells compared to untreated cells. Data shown represent the mean ± standard of error from triplicate experiments. Using Student’s *t* test, *P* value < 0.05 was considered statistically significant

**Fig. 8:**
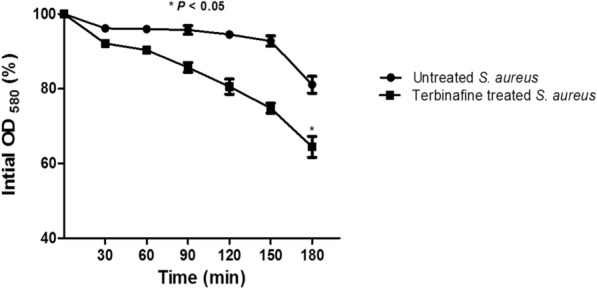
Terbinafine treated cells were more prone to Triton X-100 induced autolysis. Data shown represent the mean ± standard of error from triplicate experiments. Using Student’s *t* test, *P* value < 0.05 was considered statistically significant

In comparison with untreated *S. aureus*, terbinafine treated bacteria were highly dispersed and did not show any obvious aggregates (Fig. 7A). Additionally, both the hydrophobicity index and EPS production were significantly disrupted in terbinafine treated *S. aureus* cells as compared to untreated cells (Fig. 7B, C, respectively). Moreover, terbinafine treated *S. aureus* was more susceptible to induced autolysis than untreated bacteria. The initial optical density was significantly reduced to 64% ± 2.8 in terbinafine treated cells vs. 81% ± 2.2 in untreated cells (Fig. 8).

### Terbinafine does not interfere with DNase, esterase, or lipase activity

The influence of terbinafine treatment on *S. aureus* virulence enzymes, DNase, esterase, or lipase has been characterized. Both terbinafine-treated and untreated *S. aureus* exhibited clearance zones with equal diameters, indicating no change in DNase activity. Similarly, both terbinafine-treated and untreated *S. aureus* showed white precipitation zones with no difference in diameters indicating no significant effect on either esterase or lipase activity upon treatment of *S. aureus* with terbinafine (Data is not shown).

### Terbinafine alters the expression of* S. aureus *virulence and biofilm genes

**Fig. 9:**
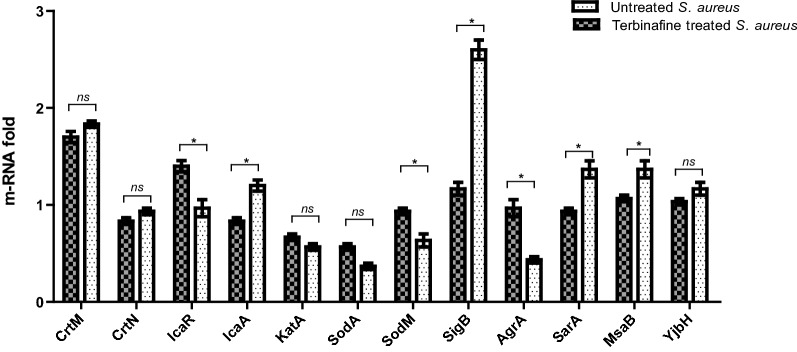
Transcriptional profile of *S. aureus* virulence genes upon terbinafine treatment. Quantitative RT-PCR revealed decreased expression of biofilm related genes and virulence regulators in terbinafine treated *S. aureus* relative to untreated bacteria. The data shown are the means ± standard errors from triplicate experiments with three technical replicates each. Using Student’s *t* test, *P* value < 0.05 was considered statistically significant

Quantitative RT-PCR showed that terbinafine does not affect the expression of staphyloxanthin biosynthesis genes, *crtM* and *crtN*. However, the expression of biofilm related genes such as *sarA* and *icaA* was significantly down-regulated and *agr A* was upregulated in *S. aureus* upon terbinafine treatment. Similarly, the expression of transcription regulators *msaB* and *sig B* was significantly repressed in terbinafine-treated *S. aureus* cells compared to untreated cells. On the other hand, the oxidative stress responsive gene (*sodM*) was upregulated in *S. aureus* following exposure to terbinafine (Fig. 9).

### Terbinafine synergizes antibiotics against* S. aureus*

The synergistic inhibitory potential of terbinafine with commonly used antibiotics as ampicillin, cefotaxime, azithromycin, ciprofloxacin, and gentamicin against *S. aureus* was determined using the checkerboard assay. Importantly, terbinafine showed a distinct synergistic effect in combination with all tested antibiotics, with FICIs ranging from 0.14 to 0.31 (Table 2).

**Table 2:** Fractional inhibitory concentration index (FICI) of terbinafine and tested antibiotics against *S. aureus* ATCC 6538

Agent	MIC (µg∕mL)		FIC	FICI	Interpretation
	Alone	Combination			
Terbinafine	2624	20.5	0.08	0.14	S
Ampicillin	8	1	0.13		
Terbinafine	2624	164	0.06	0.31	S
Cefotaxime	8	2	0.25		
Terbinafine	2624	41	0.02	0.15	S
Azithromycin	1	0.125	0.13		
Terbinafine	2624	41	0.02	0.15	S
Ciprofloxacin	2	0.25	0.13		
Terbinafine	2624	82	0.03	0.16	S
Gentamycin	2	0.25	0.13		

*FIC* Fractional inhibitory concentration, *FICI* fractional inhibitory concentration index, *S* synergism

### Terbinafine weakens* S. aureus *pathogenesis in vivo

**Fig. 10:**
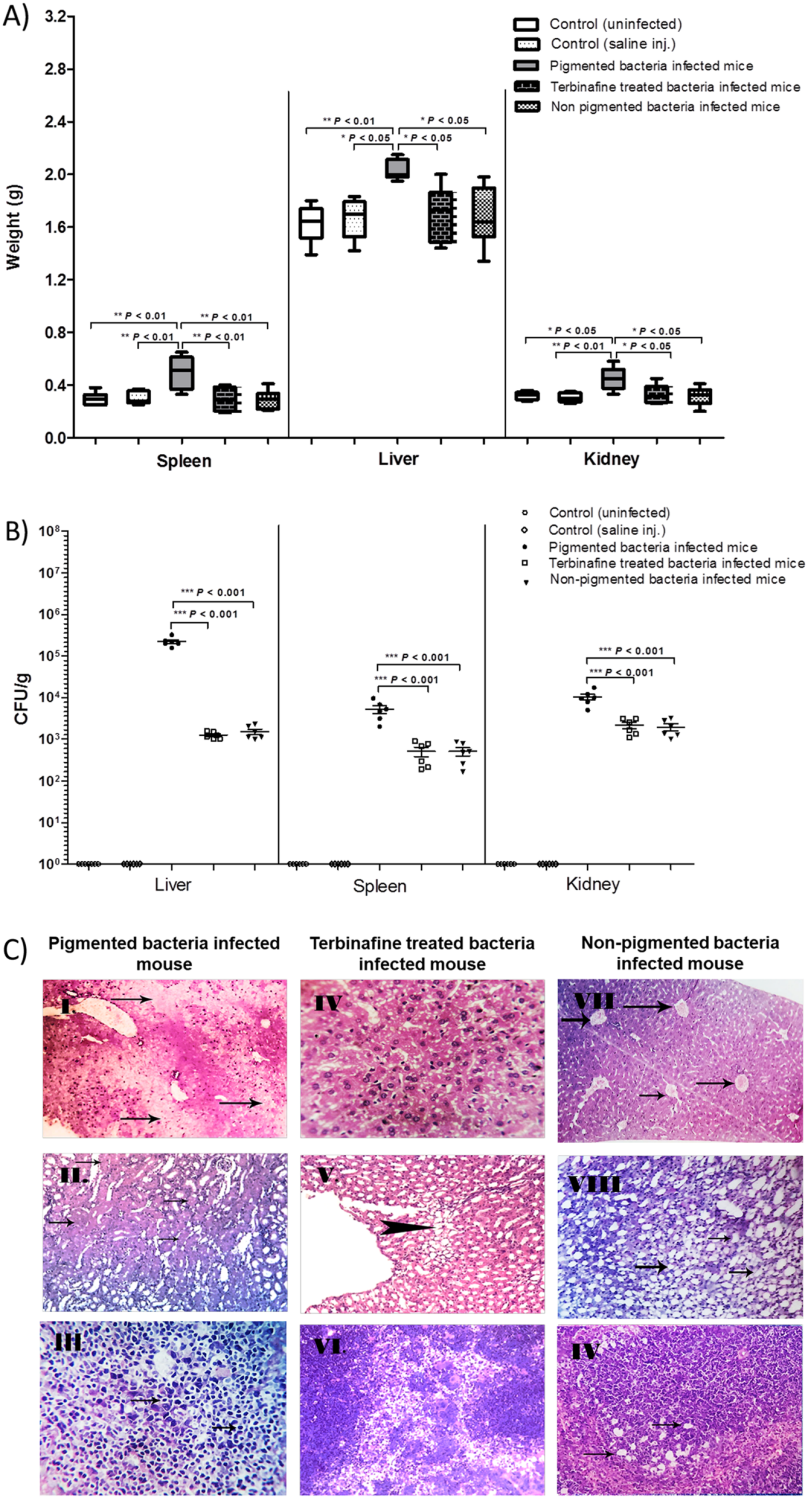
Terbinafine altered *S. aureus* mice pathogenesis. **A** Increase in organ weight of pigmented bacteria inoculated mice compared to terbinafine treated and non-pigmented inoculated mice. **B** Bacterial load of liver, spleen and kidney of each group. **C** Histopathological organ section from pigmented, terbinafine treated and non-pigmented isolate stained by hematoxylin and eosin stain. (I) Liver with diffuse areas of caseous necrosis. (II) Degeneration of some renal tubules represented in cloudy swelling (III) Focal necrosis of some spleen lymphocytes in the white pulp. (IV) Normal hepatic parenchyma with normal tissue architecture and cellular details. (V) Focal cystic dilation of some renal tubules (arrowhead). (VI) Spleen with depleted white pulp lymphocytes. (VII) Hepatic blood vessels with diffuse congestion (arrows) and dilated sinusoids. (VIII) Kidney with diffused cystic dilation of some renal tubules in the renal medulla. (IX) Spleen with focal vacuolar degeneration. Each symbol represents the value for an individual mouse and horizontal bars indicate the means**.** Using Mann–Whitney *U* analysis*, P* value < 0.05 was considered statistically significant

Mice were infected with *S. aureus* (pigmented and terbinafine treated bacteria), and signs of bacterial infection were compared to mice injected with non-pigmented and untreated bacteria. Spleen, liver, and kidney isolated from mice infected with pigmented *S. aureus* were congested, exhibiting a significant increase in weight as compared to those mice injected with either terbinafine-treated or non-pigmented bacteria (Fig. 10A). Furthermore, the numbers of colonizing bacteria were determined in organs isolated from infected mice. Importantly, pigmented *S. aureus* colonized more significantly mice organs; spleen, liver, and kidney (5255 ± 11, 222,492 ± 23, 10,297 ± 16 CFUs/g, respectively) as compared to both terbinafine treated bacteria (506 ± 13, 1245 ± 11, 2137 ± 363.3 CFUs/g, respectively) and non-pigmented *S. aureus* (507 ± 11, 1506 ± 22, 1938 ± 362.8 CFUs/g, respectively) (Fig. 10B). Additionally, histopathological examination of organ sections isolated from mice infected with pigmented *S. aureus* revealed tissue inflammatory responses with irreversible tissue necrosis and fibrosis. On contrast, organs isolated from mice inoculated with either terbinafine treated or non-pigmented *S. aureus* showed normal tissues with minimal pathological changes (Fig. 10C).

## Discussion

*Staphylococcus aureus* is a leading pathogen causing infections with high morbidity and mortality rates that impose a heavy financial burden on hospitals worldwide [1]. Antibiotic-resistant *S. aureus* is on the rise, highlighting the need for novel therapeutic strategies. Thus, introducing of antimicrobials that hinder *S. aureus* virulence without impeding bacterial growth has been proposed [5]. The current study shows, for the first time, fully detailed evidence for terbinafine anti-virulent activity against *S. aureus*. Our findings clearly demonstrate that terbinafine alleviates *S. aureus* staphyloxanthin production, biofilm formation, as well as host pathogenesis.

Staphyloxanthin is a carotenoid pigment that functions as an antioxidant, mediating bacterial survival under oxidative stress and is involved in host pathogenesis and disease progression [28]. Present data demonstrates that terbinafine possesses a dose dependent inhibition of staphyloxanthin in *S. aureus*. To precisely unveil the molecular mechanism of terbinafine, staphyloxanthin biosynthesis intermediates were extracted and quantified from *S. aureus* following treatment with terbinafine. Interestingly, terbinafine treatment led to the accumulation of CrtN substrates, 4, 4′-diapophytoene, and a low abundance of the subsequent intermediates, suggesting interference of terbinafine with CrtN. In line with current results, Chen et al. [19] reported that naftifine, an allylamine antifungal, has pigment inhibitory activity through targeting CrtN and attenuates *S. aureus* virulence. Importantly, qRT-PCR analysis indicates that terbinafine has no effect on the expression of either *crtM* or *crtN*. On the other hand, the molecular docking study performed herein validated terbinafine interaction with CrtN. Similarly, Ye et al. [51] has reported that a novel cationic peptide MSI-1 significantly inhibited staphyloxanthin production through binding with CrtN without any change in the expression of *crtM* or *crtN.* The molecular docking results strongly indicate that terbinafine inhibitory activity on staphyloxanthin is likely to be due to direct interference with CrtN enzyme activity.

As a consequence of staphyloxanthin inhibition, staphyloxanthin induced protective activity was disrupted and terbinafine treated *S. aureus* was more susceptible to both oxidative and acidic stress relative to untreated bacteria [28]. This higher susceptibility to stressful conditions suggests that *S. aureus* would be more sensitive to reactive oxygen species and acidic pH acting inside the phagolysosome, limiting *S. aureus* intracellular survival [52, 53]. Furthermore, terbinafine sensitized *S. aureus* cells to human blood cells, which could result in increased immune clearance by the host. In addition to inhibition of staphyloxanthin biosynthesis, the increased sensitization of *S. aureus* to stressful conditions upon terbinafine treatment could be attributed to the down regulation of *sigB* and *msaB*, which have been reported to be involved in *S. aureus* survival under various stresses [43, 54]. Pandely et al*.* demonstrated that *msaABCR* operon regulates an oxidative stress defense mechanism facilitating the persistence and recurrence of staphylococcal infections [55]. Accordingly, terbinafine would be valuable to tackle recurrent and persistent staphylococcal infections.

The decrease in *S. aureus* membrane fluidity has been linked to membrane staphyloxanthin content, elucidating the role of staphyloxanthin in regulating membrane stiffness [56]. FTIR analysis has been recently utilized for studying *S. aureus* membrane biophysical characters [57]. In the current study, the FTIR spectrum of terbinafine treated *S. aureus* showed changes in membrane lipids and polysaccharides comparable to untreated cells [29]. Since *S. aureus* has intrinsic resistance to membrane-targeting antibiotics, the altered biophysical properties of the bacterial membrane rendered terbinafine treated *S. aureus* more susceptible to polymxin B killing. Accordingly, Valliammai reported that thymol efficiently inhibited staphyloxanthin biosynthesis and senstizied *S. aureus* cells to polymyxin B [30]. Thus, these findings could suggest the use of staphyloxanthin inhibitors such as terbinafine in concurrent with membrane targeting antibiotics to offset *S. aureus* resistance.

The propensity to form biofilm is considered to be a chief virulence factor of several pathogens, including *S. aureus*, resulting in persistent and recurrent infections within the host [12]. *S. aureus* commonly contaminates indwelling medical devices. *S. aureus* is capable of forming biofilms on polystyrene and polypropylene plastic polymers that are routinely used in the manufacture of medical devices [13]. In the present study, terbinafine significantly reduced *S. aureus* ability to form biofilm on both polystyrene and polypropylene surfaces in a dose dependent manner. The naphthalene moiety incorporation has recently reported by Ghameshlouei et al. [58] to improve biofilm inhibitory activity of oxadiazole derivatives. Terbinafine antibiofilm activity can be explained by its repressor effect on *sarA* and *msaB*, which have been strongly implicated in *S. aureus* biofilm formation. Interestingly, the mutation of *sarA* and absence of *msaABCR* in *S. aureus* were found to limit biofilm formation and lead to defect in biofilm maturation, respectively [59, 60].

Among bacterial biofilms, bacterial cell auto-aggregation and surface hydrophobicity play a major role in the initial steps of biofilm formation. Current results demonstrate a reduction in both *S. aureus* auto-aggregation and surface hydrophobicity upon terbinafine treatment. This reduction in bacterial auto-aggregation would be advantageous as *S. aureus* auto-aggregation has been linked to the increased pathogenicity, drug resistance, and host immune evasion [61]. Sarker et al. [62] also reported that reduction in cell surface hydrophobicity could hinder microbial colonization of hydrophobic surfaces and, consequently, biofilm development. Furthermore, EPS serves as a barrier to shield bacteria from antimicrobial agents and increases biofilm resistance to mechanical forces such as fluid flow and shear stress inside the catheter [63]. EPS is a polysaccharide intercellular adhesin (PIA), which is encoded by the *icaADBC* operon. Terbinafine treatment showed a dose dependent reduction in EPS content and, subsequently, in biofilm buildup and maturation. These findings were further validated by the repression of *icaA*, *sigB*, and *sarA,* which play a role in PIA biosynthesis. In addition to the induction of *icaR*, which binds to DNA region upstream *icaA,* causing suppression of *icaADBC* operon transcription [64]. Notably, Yazdani et al. [65] and Fowler et al. [66] have found an association between expression of *ica* genes and biofilm formation ability.

Combination therapy has been introduced to overcome bacterial resistance to conventional antibiotics [67]. Upon terbinafine treatment*, S. aureus* exhibited an increased rate of cell death. This increased bacterial autolytic activity could be beneficial in potentiating the bactericidal activity of cell wall-active antibiotics [68]. The increase in *S. aureus* induced autolysis was explained by repression of *msaB*, which has been recently reported to regulate the rate of cell death [69]. Accordingly, the current data show a synergistic effect between terbinafine and β-lactam antibiotics ampicillin and cefotaxime. This synergism could be attributed to the functional membrane microdomain disassembly as a result of staphyloxanthin inhibition by terbinafine, which would interfere with PBP2a oligmerization, rendering *S. aureus* more susceptible to penicillin [70]. Moreover, *msaB* has been reported to control peptidoglycan cross linking, affecting *S. aureus* susceptibility to cell wall acting antibiotics like β-lactams. Antibiotics such as ciprofloxacin and gentamicin alter bacterial cellular respiration, resulting in a lethal level of intracellular damaging reactive species [71]. Therefore, the combination of terbinafine with ciprofloxacin or gentamicin would sensitize *S. aureus* to oxidative stress generated by both immune cells and antibiotics. Interestingly, Márió Gajdács has recently reported that terbinafine could be used as adjuvant as it efficiently reduced MIC value of ciprofloxacin [72]. These results clearly suggest the use of terbinafine as an adjuvant in combination therapy to overcome *S. aureus* resistance to conventional antibiotics.

Staphyloxanthin has been found to have a substantial role in host pathogenesis. The effect of terbinafine on *S. aureus* virulence was evaluated in vivo using mice infection model. Interestingly, terbinafine treated *S. aureus* exhibited a weakened virulence potential, causing fewer serious and reversible lesions compared to untreated *S. aureus*. This is in line with Chen et al*.* [19], who reported that naftafine attenuated *S. aureus* virulence in mouse infection models. This attenuated virulence could attributed to staphyloxanthin inhibition that rendered *S. aureus* more susceptible to neutrophils killing and innate immune inactivation in mouse infection model [73]. Additionally, Blevins et al., revealed that mutations in the virulence regulatory genes *sigB* and/or *sarA* reduced *S. aureus* capacity to cause in vivo pathogenesis [74, 75]. It has been shown that SigB plays an important role in *S. aureus* acclimation during chronic infections [76].

## Conclusion

The current study demonstrates the anti-virulence potential of terbinafine against *S. aureus*. Terbinafine revealed significant anti-staphyloxanthin activity, sensitizing *S. aureus* to stressful conditions and host killing. Staphyloxanthin inhibition by terbinafine disrupted cell membrane permeability, leading to increased sensitivity to membrane targeting antibiotics. Additionally, terbinafine exhibited a dose dependent inhibition of *S. aureus* biofilm formation through interfering with the several stages of biofilm formation regarding cell autoaggregation, cell surface hydrophobicity, and EPS production. Interestingly, terbinafine synergized the activity of conventional antibiotics and alleviated *S. aureus* pathogenesis in the host. Terbinafine anti-virulent activity against *S. aureus* is multifactorial. In addition to its anti-pigment potential, terbinafine represses global virulence regulators in *S. aureus*, such as MsaB*,* SarA, and SigB. The findings of the current study are valuable and highlight the importance of terbinafine in the management of *S. aureus* infections, providing evidence that terbinafine could be repurposed as an anti-virulent agent against *S. aureus*.

## Data Availability

The authors confirm that the data supporting the findings of this study are available within the article. **Ethics approval and consent to participate** Experiments involving human participants were performed in accordance to the Declaration of Helsinki and approved by Zagazig University Institutional Review Board (ZU-IRB). All procedures in the animal study section were carried out in accordance with the ethical standards of the Zagazig University-Institutional Animal and Use Committee (ZU-IACUC), which was granted approval number (ZU-IACUC/3/F/158/2019).

## Abbreviations

MRSA: Methicillin-resistant *S. aureus*
VRSA: Vancomycin-resistant
PIA: Polysaccharide intracellular adhesion
ica: Intracellular adhesion
TSB: Trypticase soya broth
MIC: Minimum inhibitory concentration
MH: Muller Hinton
PBS: Phosphate buffered saline
TSA: Trypticase soya agar
MOE: Molecular operating environment
CFUs: Colony forming units
FTIR: Fourier infrared spectroscopy
CV: Crystal violet
SEM: Scanning electron microscopy
HI: Hydrophobicity index
EPS: Exopolysaccharide
DNase: Deoxyribonuclease
FICI: Fractional inhibitory concentration index
